# A Regression-Based Framework for Quantitative Assessment of Muscle Spasticity Using Combined EMG and Inertial Data From Wearable Sensors

**DOI:** 10.3389/fnins.2019.00398

**Published:** 2019-05-03

**Authors:** Xu Zhang, Xiao Tang, Xiaofei Zhu, Xiaoping Gao, Xiang Chen, Xun Chen

**Affiliations:** ^1^Department of Electronic Science and Technology, University of Science and Technology of China, Hefei, China; ^2^Department of Rehabilitation Medicine, First Affiliated Hospital of Anhui Medical University, Hefei, China

**Keywords:** spasticity assessment, machine learning, surface electromyogram, stroke, home-based rehabilitation

## Abstract

There have always been practical demands for objective and accurate assessment of muscle spasticity beyond its clinical routine. A novel regression-based framework for quantitative assessment of muscle spasticity is proposed in this paper using wearable surface electromyogram (EMG) and inertial sensors combined with a simple examination procedure. Sixteen subjects with elbow flexor or extensor (i.e., biceps brachii muscle or triceps brachii muscle) spasticity and eight healthy subjects were recruited for the study. The EMG and inertial data were recorded from each subject when a series of passive elbow stretches with different stretch velocities were conducted. In the proposed framework, both lambda model and kinematic model were constructed from the recorded data, and biomarkers were extracted respectively from the two models to describe the neurogenic component and biomechanical component of the muscle spasticity, respectively. Subsequently, three evaluation methods using supervised machine learning algorithms including single-/multi-variable linear regression and support vector regression (SVR) were applied to calibrate biomarkers from each single model or combination of two models into evaluation scores. Each of these evaluation scores can be regarded as a prediction of the modified Ashworth scale (MAS) grade for spasticity assessment with the same meaning and clinical interpretation. In order to validate performance of three proposed methods within the framework, a 24-fold leave-one-out cross validation was conducted for all subjects. Both methods with each individual model achieved satisfactory performance, with low mean square error (MSE, 0.14 and 0.47) between the resultant evaluation score and the MAS. By contrast, the method using SVR to fuse biomarkers from both models outperformed other two methods with the lowest MSE at 0.059. The experimental results demonstrated the usability and feasibility of the proposed framework, and it provides an objective, quantitative and convenient solution to spasticity assessment, suitable for clinical, community, and home-based rehabilitation.

## Introduction

Spasticity is a clinical symptom of neural disorders and was prevalent in stroke, spinal cord injury, cerebral palsy and multiple sclerosis patients (Nielsen et al., 2007; Naghdi et al., 2014). It was considered to be caused by the increased excitability of stretch reflex (Nielsen et al., 2007). The primary manifestation of spasticity is an abnormal muscle tone in the involved muscle, which is generally shown as a resistance when the muscle is stretched passively (Trompetto et al., 2014). The resistance is also found to be velocity-dependent (Lee et al., 2002; Mullick et al., 2013). The spasticity may cause abnormal physical appearance and even muscle contracture (Ada et al., 2006; Naghdi et al., 2014), consequently weakening the ability of physical activity and seriously affecting the daily life of patients. Therefore, quantitative assessment of spasticity is vital for early interventions during rehabilitation treatment.

For quantitative assessment of spasticity, routine clinical scales such as modified Ashworth scale (MAS) and the modified tardieu scale (MTS) are most frequently used (Mehrholz et al., 2005). The MAS measures the resistance during passive soft-tissue stretching without consideration of stretch velocity. According to the specified regulations pre-defined in MAS, clinicians are able to grade the spasticity. Although limitations in performance exist due to its rough measurement procedures and relative subjectivity (Pandyan et al., 1999, 2003; Fleuren et al., 2010; Phadke et al., 2015), standardized user guidelines have been developed and the inter-rater reliability for testing of upper limb spasticity has been proven to be acceptable (Blackburn et al., 2002). In addition, due to its convenience without requirement of instrument, the MAS is a primary clinical measure of spasticity and is even considered as a gold standard of being the referential yardstick for other new spasticity measurement methods (Pisano et al., 2000). The measurement of MTS involves two passive movements at a very slow speed and a high speed as fast as possible, respectively (Mehrholz et al., 2005). It has been regarded as a more reliable scale for spasticity assessment with higher inter-rater reliability demonstrated in several studies (Singh et al., 2011), but the validity and reliability of MTS are still doubtable in some investigations (Mehrholz et al., 2005; Naghdi et al., 2014). With this semi-quantitative manner of measurement, the use of clinical scales just meets basic needs of clinical applications. More objective and quantifiable tools are demanded for muscle spasticity assessment.

Electrophysiological techniques can be used to examine altered neurotransmission in the spinal neuronal pathway for understanding underlying pathophysiology as well as assessing muscle spasticity (Pisano et al., 2000; Cooper et al., 2005; Biering-Sørensen et al., 2006; Kim et al., 2011; Bar-On et al., 2013; Naghdi et al., 2014). Both Hoffmann-reflex (H-reflex) test and electromyography (EMG) measurement are always involved in these techniques. Several investigations reported that biomarkers extracted from the H-reflex test increased in patients with spasticity compared to healthy controls but such an increase exhibited no or poor correlation to the clinical scores (Naghdi et al., 2014). An increase in EMG biomarker during instrumented passive stretch was found to moderately correlate with the MAS grades in lower limb spasticity of children with cerebral palsy (Jobin and Levin, 2000; Bar-On et al., 2013). Despite limited contribution to the quantitative assessment of spasticity, the electrophysiological examinations can provide an easy and the most reliable way of determining the stretch reflex threshold and information concerning neural transmission. Thus their potential role in quantifying spasticity is significant.

Alternatively, biomechanical approaches are frequently used to quantify the velocity-dependent resistance during passive movement by measuring joint position, angular velocity, and torque (reactive-resistance) (Pisano et al., 2000; Condliffe et al., 2005; Ardabili et al., 2011; Bar-On et al., 2013; Li et al., 2017). These investigations focus on assessing the muscular mechanics of spasticity, and have achieved highly reliable assessment for spasticity. For example, McGibbon et al. (2016) presented a kinematic model and addressed the issue of identifying categorical degrees of muscle spasticity by classification learning using a linear discriminant analysis algorithm. However, specific devices such as the isokinetic and torque sensors and well-controlled laboratory environments are generally required to ensure precise measurement and accurate quantification (Condliffe et al., 2005; Ardabili et al., 2011). With sophisticated protocols, biomechanical approaches are usually involved in scientific research rather than clinical practice.

Furthermore, various investigations about spasticity have mentioned that the spasticity resistance consists of both neurogenic and non-neurogenic (primarily mechanical) components (Huang et al., 2004; Lam et al., 2005). The former mainly represents abnormal neural regulation and induced hyperactivity of motoneuron (Nielsen et al., 2007). The latter may result from altered mechanical properties of muscle soft tissues (the muscle fibers and tendons) and limb joints, which form the secondary contributor to the spasticity resistance (Stecco et al., 2014). Thus it is reasonable that a precise spasticity assessment would involve both electrophysiological and biomechanical aspects. Recently, many efforts have been made toward assessing spasticity from both aspects with simultaneous use of electrophysiological and biomechanical measurements. For example, NeuroFlexor has been developed by Lindberg et al. (2011) as a fine-tuned device with capability of quantitative discrimination between the neural and mechanical components of the total resistance as a result of muscle spasticity. Due to its capability, this device was reported to be used for spasticity assessment with satisfactory precision and reliability.

Besides the precision, there is a great demand for wearability and portability of the devices and fast and convenient implementation of the measurement. Spasticity is always regarded to vary across individuals and to last a long term over multiple stages of motor recovery. Its assessment is required to be repeated for continuous management in multiple sites from inpatient clinic through community to home. Specifically, considering the prevalence of muscle spasticity after a variety of neurological injuries, there is an increasing demands for implementing spasticity assessment in community and home environments, where it is impractical to apply high-cost specialized instruments and complex experimental setups. Therefore, it is of great significance to develop a convenient and effective approach for quantitative assessment of muscle spasticity suitable for community and home rehabilitation.

With the above-mentioned considerations, this paper presents a novel regression-based framework for quantitative assessment of muscle spasticity using a wearable sensing system including surface EMG (sEMG) and inertial sensors along with a simple and practical procedure. Corresponding to both the neural and biomechanical components contributing to muscle spasticity, two models (the lambda model and the kinematic model) are built respectively to derive biomarkers from sensory data, which are discriminable across different degrees of spasticity. These biomarkers, from one model or the combination of both models need to be processed to predict an evaluation score that is the same meaningful as the routine clinical assessment score (i.e., the MAS) but has purely objective and quantitative characters. Supervised regression learning is a nature solution as it is able to establish an expert system (also known as numerical model) for automatically and accurately producing numerical evaluation scores. Therefore, supervised regression algorithms such as single-/multi-variable linear regression and support vector regression (SVR) were employed within the framework. The framework proposed in this study provides a practical solution to spasticity assessment in a convenient, objective and quantitative way. With the demonstrated effectiveness, such convenience can especially extend the sites for assessing the muscle spasticity and its intervention outcome from inpatient hospital to home, toward advanced spasticity management and rehabilitation.

## Materials and Methods

### Subjects

Sixteen subjects with spasticity (14 males and 2 females; aged from 33 to 71 years, averaged 54 ± 10 years; time duration since diagnosis from 19 to 86 days, averaged 52 ± 10 days) and eight healthy subjects (six males and two females; aged from 22 to 45 years, averaged 29 ± 9 years) were recruited in the study. This study was approved by the Ethics Review Committee for Clinical Medical Research of First Affiliated Hospital of Anhui Medical University. Inclusion criteria for participants with spasticity include: (a) currently experiencing one of the following disease: stroke (cerebral ischemia, brain hemorrhage or brainstem infarction and etc.), acquired brain trauma or incomplete spinal cord injury and accompanied by spasticity in flexor and extension muscles of the elbow; (b) the spasticity of elbow extensor or flexor was assessed within 1–3 grades using MAS; (c) the range of elbow joint during passive stretch was at least 120 degrees; (d) medically stable with clearance to participate; (e) without any historical musculoskeletal injuries or cognition problems; (f) able to offer informed signed consent prior to any procedure of the experiment. For all subjects, clinical assessment of spasticity using the MAS was performed just half an hour prior to their participation into the experiment, by one qualified, experienced physical therapist. The demographic information of the participants with spasticity is presented in Table 1, and characteristics of healthy subjects is provided in Table 2.

**Table 1:** Demographic information for each of 16 subjects with muscle spasticity.

No.	Age range	Sex	Brain lesion	Involved muscle	Duration (days)	Spastic side	MAS grade
1	46–50	Male	Brain hemorrhage	BB	56	Left	1+
2	41–45	Male	Brainstem hemorrhage	TB	69	Left	2
3	46–50	Male	Brain hemorrhage	TB	38	Right	1
4	71–75	Male	Brain hemorrhage	BB	29	Right	1+
5	56–60	Male	Brain infarction	BB	52	Right	1+
6	56–60	Female	Brain infarction	BB	76	Left	2
7	56–60	Male	Brain infarction	BB	73	Left	1+
8	56–60	Male	Left thalamic hemorrhage	TB	37	Right	2
9	46–50	Male	Brain hemorrhage	TB	62	Right	3
10	41–45	Male	Spinal cord injury	BB	86	Left	1
11	61–65	Female	Brain hemorrhage	BB	19	Right	1
12	56–60	Male	Brain hemorrhage	BB	52	Left	2
13	31–35	Male	Brain hemorrhage	BB	47	Right	1+
14	61–65	Male	Acquired brain trauma	BB	38	Right	1
15	46–50	Male	Brain hemorrhage	BB	60	Right	1+
16	36–40	Male	Spinal cord injury	BB	35	Right	3

*BB, biceps brachii muscle; TB, triceps brachii muscle; MAS, modified Ashworth scale.*

**Table 2:** Demographic information for each of eight healthy subjects.

No.	Age range	Sex	Tested side	MAS grade
1	25–30	Female	Right	0
2	41–45	Male	Left	0
3	21–25	Male	Left	0
4	21–25	Male	Left	0
5	21–25	Male	Right	0
6	21–25	Female	Left	0
7	21–25	Male	Left	0
8	36–40	Male	Left	0

*MAS, modified Ashworth scale.*

### Experiments

**FIGURE 1:**
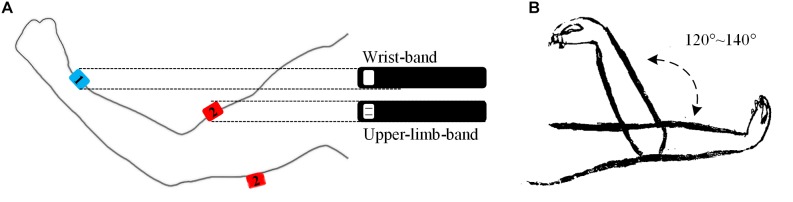
Illustrations of the band-like sensor placement **(A)** and a passive elbow stretch **(B)** in the experiment. The sensor marked in number 1 stands for the IMU and the sensor marked in 2 a sEMG sensor, which was embedded in a wrist band and an upper-limb-band, respectively. The sEMG sensor can be used to target at the biceps brachii muscle or the triceps brachii muscle respectively.

A home-made multi-channel signal recording system supporting up to 16 sEMG recording channels and 9-axis inertial measurement units (IMUs) was used in this study. In this system, each individual EMG sensor consists of two parallel bar-shaped electrodes with a width of 1 mm, a length of 10 mm, and a between-electrode distance of 10 mm, thus constituting one single-differential sEMG recording channel. Each IMU (MPU9250, InvenSense, San Jose, CA, United States) consisted of a 3-axis accelerometer, a 3-axis gyroscope and a 3-axis magnetometer for recording inertial data. In the experiments, one sEMG sensor was used to target at the belly of the biceps brachii muscle or the triceps brachii muscle (depending on appearance of the muscle spasticity) with its electrode bar perpendicular to the muscle fibers and the IMU was placed on the ipsilateral medial wrist for recording stretch angular velocity (see Figure 1A). The surface EMG sensor and the IMU were embedded in a stretchable armband and wristband respectively to ensure secured placement and to enhance portability of both sensors. A round reference electrode (Dermatrode; American Imex, Irvine, CA, United States) was placed on arm fossa cubitalis on the same side. In this system, each sEMG channel was amplified with a gain of 600 in total and further digitized by a 16 bit anolog-to-digital converter (ADS1198, Texas Instruments, Dallas, TX, United States) with a sampling rate of 1 kHz. The IMU was designed to produce digitalized data at 100 Hz per axis. The digitalized data from both sensors were simultaneously recorded.

After skin preparation with medical alcohol, the subjects were asked to participate into two sessions of test. Each session consisted of 15 to 20 trials of passive stretch with different velocities. In a single trial, the subject was instructed to fully extend his tested elbow at 180 degrees with palm upward. Then, the tested elbow was passively pulled by an experimenter to elbow flexion at 40–60 degrees with a stretch range of 120–140 degrees. After a 2-s pause, it was passively stretched back to 180 degrees (see Figure 1B). In each trial, the stretch velocity was determined subjectively by the experimenter and kept to be almost constant during the stretch. Varying stretch velocities were applied among the 15–20 trials, thus resulting in a varying duration of 3–7 s for each trial. Sufficiently long resting periods were allowed between any two consecutive trials and sessions to avoid mental or muscular fatigue and to reduce stretch-induced temporary muscle tones. No specialized equipment was required to control and record the velocity or stretch angle directly in this experiment. Meanwhile, all data were recorded by the system, transferred and restored to a portable computer via a USB cable for further analysis.

### Data Analysis

**FIGURE 2:**
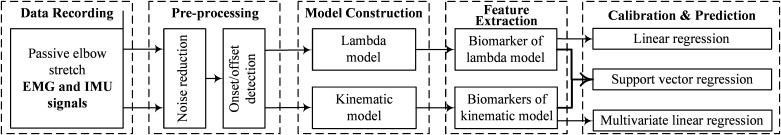
Block diagram of the proposed framework for spasticity evaluation.

The data analysis procedure of the proposed regression learning framework for spasticity evaluation is shown in Figure 2, with details described as follows.

#### Data Pre-processing and Segmentation

**FIGURE 3:**
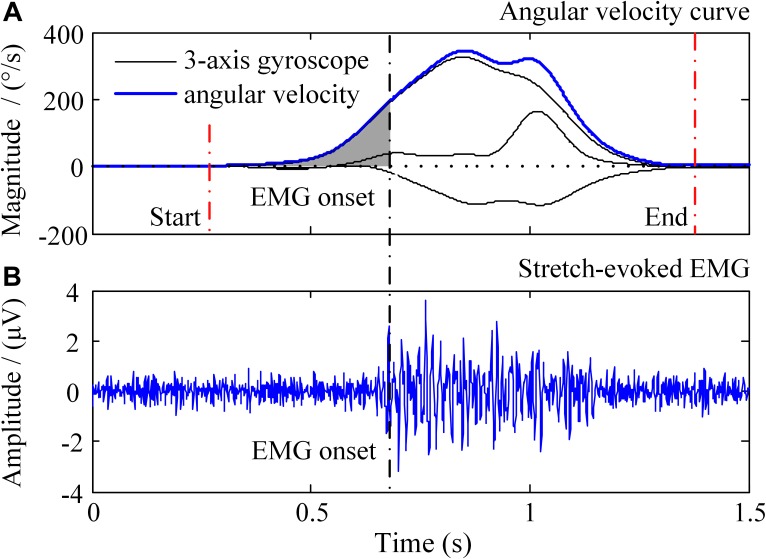
Illustration of the recorded signals in a trial of passive elbow stretch. In the **(A)**, the black solid lines represent 3-axis gyroscope data and the blue solid line is the angular velocity, respectively. The **(B)** shows the sEMG signals evoked by passive elbow stretch.

All the data were processed in the Matlab (version, 2014a, The Mathworks, Inc., Natick, MA, United States). The sEMG signals were filtered by a zero-lag fourth-order Butterworth band pass filter set at 20–450 Hz. Since only the angular velocity data were used in this study, the gyroscope signals were selected and then filtered by a zero-lag second-order Butterworth low pass filter set at 10 Hz. Representative recorded signals of a passive stretch trial were showed in Figure 3, including a three-axis gyroscope signal and an EMG signal. The stretch angular velocity was the vector superposition of the three-axis gyroscope signal. An amplitude thresholding method was employed to detect the onset of sEMG response as well as the onset and offset of stretch movement (Silva et al., 2017), as the vertical dashed lines marked in Figure 3, and its threshold was set at three times standard deviation of the baseline (Zhang et al., 2011). After auto-detection of onset, those segments with obvious noise contamination were discarded by visual examination.

#### Lambda Model

The lambda model is a classic measure to quantitatively evaluate spasticity using surface EMG and motion data (Levin et al., 2000). This model evaluates the stretch reflex threshold which refers to the joint angle when the EMG signal is exactly induced during a stretch movement of a targeted muscle (Levin et al., 2000). In addition, it presents that the regulation of the central nervous system (CNS) for the stretch reflex threshold is an essential mechanism for joint motion control, and it is related to the control of joint position. Thus the lesion of CNS after stroke may interferes with the regulation process then result in a deficits of the stretch reflex threshold regulation (Levin et al., 2000; Calota et al., 2008). As a result, the induced timing of EMG could vary a lot due to different degrees of CNS lesions. This could be reflected in the model in terms of decreased stretch reflex threshold. A velocity-dependent character of the stretch reflex threshold is also demonstrated in this model, for it is shown by the fact that a higher stretch velocity leads to a shorter latency of the stretch reflex. The shorter latency can be represented by a smaller angle range for joint motion.

**FIGURE 4:**
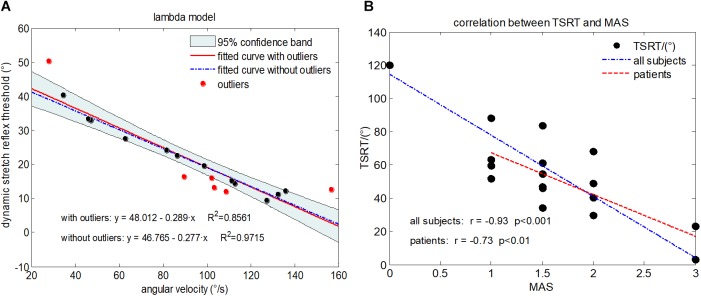
**(A)** Illustration of the lambda model constructed for a representative subject (Subject 1) with MAS grade of 1+, where each data point is derived from a trial. The regression line derived from all data points is shown in red. The data points in red represent outliers. The final regression line was represented by a blue dashed line when outliners were excluded. **(B)** The scatter plot showing relationship between the TSRT value and MAS grades for all subjects. Each subject is represented by a data point located by its TSRT value and MAS grade. The blue dashed line is derived from regression analysis based on data of all subjects including healthy controls and subjects with spasticity. The red line denotes regression analysis performed on only subjects with spasticity.

Considering that the increase of stretch angular velocity generally gives a decreasing trend of stretch reflex threshold, a linear regression model was therefore built between the stretch angular velocity and dynamic stretch reflex threshold (DSRT, refers to the mutative stretch reflex threshold, the joint angular when EMG is induced, at different stretch angular velocity which is greater than zero) in these investigations. Please note that the original proposer accurately quantified the angular velocity with specialized equipment. With wearable IMU sensors to measure elbow joint movements, however, such velocity needed to be estimated approximately. The mean stretch angular velocity was calculated over the entire duration of elbow stretch as a rough estimation of the elbow joint stretch velocity. The DSRT was calculated by integrating the stretch angular velocity from the beginning to the moment when the EMG was evoked, which was marked as shaded area in Figure 3. Thus, the mean stretch angular velocity (independent variable) and the DSRT (dependent variable) were applied to construct the linear lambda model using a linear regression, as showed in Figure 4A. Due to the special design of approximation estimation using the averaging approach, multiple trials were required to enhance reliability of the regression analysis with a sufficient amount of data points. This explains the reason for conducting multiple trials in the experimental protocol. On this basis, exclusion of outliers was further implemented using a 95% confidence interval (Montgomery et al., 2012), when the linear regression was conducted for each subject. With the determined linear regression line, the intercept was calculated to be a special DSRT when the stretch velocity approximated into zero. It was termed tonic stretch reflex threshold (TSRT) in this study, indicating the stretch reflex threshold under quasi-static stretching. According to previous studies, the TSRT in the lambda model represents the physiological range of stretch reflex regulation (Levin et al., 2000). In general, the TSRT may exceed the biomechanical range of joint movement for healthy subjects (Musampa et al., 2007), as a reflection of normal stretch reflex regulation. For patients with spasticity, the lesion of CNS may result in a disorder of stretch reflex regulation, which would reduce the stretch reflex threshold as well as limit the range of joint movement. Therefore, TSRT was regarded as a biomarker derived from the lambda model for spasticity assessment. Please also note that sEMG would not be evoked until the stretch velocity exceeded a large threshold for healthy subjects. For any subject without typically evoked EMG in most of the trials (across multiple velocities), the TSRT was conformably set to 120 degrees, according to the physiological relevance of the lambda model.

In order to evaluate the degree of spasticity for each patient, the biomarker TSRT needs to be calibrated with a meaningful scale, which referred to the routine MAS in this study. Thus, the calibration procedure was implemented by the means of performing a supervised linear regression analysis between the TSRT biomarkers and MAS grades. In this analysis, MAS grades 0, 1, 1+, 2, and 3 were numerically represented by 0, 1, 1.5, 2, and 3, respectively. Given experimental data from 24 subjects in total (16 subjects with spasticity and 8 healthy controls), a 24-fold leave-one-out cross-validation was performed. Data from 23 subjects were used as the training dataset to establish a regression line as the best fit between their biomarkers and MAS grades (i.e., labels for supervised learning), whereas data from the remaining subject were used as the testing dataset to predict an evaluation score from the input biomarker (i.e., the input feature) based on the learnt regression line. Thus, after 24 rounds of testing, each subject had a predicted evaluation score. Such a procedure was designed also with the purpose to verify the performance of the biomarker TSRT in assessing muscle spasticity.

#### Kinematic Model

The kinematic model was proposed for spasticity evaluation by McGibbon et al. (2013), which was based on a constant-jerk assumption. According to the assumption, the intended motion curve of elbow stretch could be constructed for each subject as a reference motion pattern (Musampa et al., 2007). A consistent pattern is assumed between the actual and re-constructed (reference) motion curve across healthy control muscles, whereas this consistency cannot be found in spastic muscles. Significant deviations from the reference pattern were observed on the actual motion curve due to interference from abnormal muscle tension. Consequently, the similarity between the actual motion curve and reference pattern was used to evaluate the degree of spasticity resistance.

**FIGURE 5:**
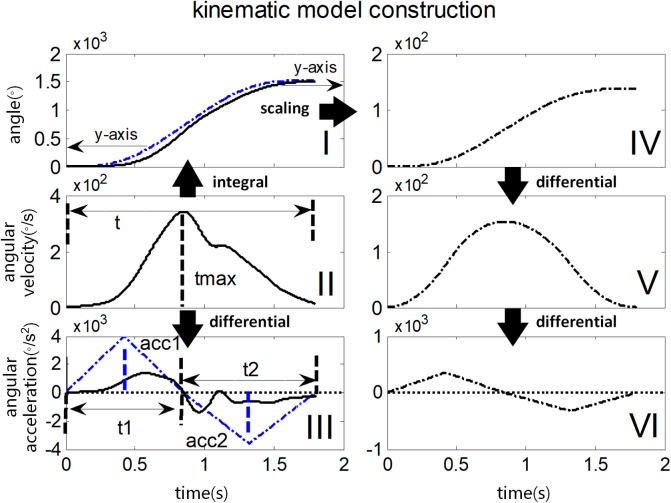
Illustration of constructing kinematic variables in the kinematic model. The black solid line in I, II, and III represents the actual angle curve, the angular velocity curve, and the angular acceleration curve respectively during passive elbow stretch. Their reconstructed and scaled curves are shown in the black dotted line in IV, V, VI on the right panel. The blue dotted line in I and III illustrates the scaling and reconstruction procedure respectively, in detail for comparison.

The construction of kinematic model is illustrated in Figure 5, with an actual example of angular velocity curve recorded from the gyroscope (marked as a black solid line in Figure 5 II) during a stretch in a single trial. Firstly, its entire time duration (t) was divided into two periods t1 and t2 by the moment of its maximal value (tmax), representing an accelerating stage and a decelerating stage respectively. Next, both integral operation and differential operation were performed on the angular velocity curve to obtain an angle curve (black solid line in I) and an angular acceleration curve (black solid line in III), respectively. Then, the angular acceleration curve (III) was reconstructed to form a subtriangular curve according to constant jerk assumption. Considering the fact that the vectorial summation of the angular velocity should be zero, the peak of angular acceleration in the second stage (denoted as acc2) was determined while this peak in the first stage (denoted as acc1) was optionally set at 4000 degree/s^2^ here. Once peaks of angular acceleration in both stages were determined, the reconstructed angular acceleration curve (blue dashed line in III) was obtained following a subtriangular rule. Afterward, two consecutive integrations were performed on the reconstructed angular acceleration curve to calculate a reconstructed angle curve (the blue dashed line in I), followed by a scaling procedure for matching the range of motion. Consequently, a reconstructed angle curve (the black dashed line in IV) was obtained. Finally, the reconstructed angle curve in IV was differentiated once and twice to produce a reconstructed angular velocity curve (V) and a reconstructed acceleration curve (VI), respectively.

It was found in this study (see section “Results”) that the reconstructed kinematic curves presented deviations from their corresponding recorded curves, and such deviations become greater in the cases of higher MAS grades. Especially, the angular acceleration curves exhibited more fluctuations with respect to its reconstructed ones for subjects with spasticity than healthy controls. In the kinematic model, therefore, three correlation coefficients between the actual curves and reconstructed curves of the angle, angular velocity and angular acceleration were calculated as three biomarkers. In addition, the median frequency (MDF) of actual angular acceleration curve (Szeto et al., 2005) was also calculated. Consequently, there are four biomarkers to measure the spasticity resistance for each subject. In order to minimize the effect of angular velocity variation as a result of manual stretch, we only chose the trails with time duration of 1–2 s for extracting biomarkers.

Similar to the quantitative evaluation method based on lambda model, the four biomarkers derived from the kinematic model also needed to be calibrated to produce a meaningful evaluation score by means of performing a supervised multivariate regression analysis (Berndt and Savin, 1977). A 24-fold leave-one-out cross-validation was applied to data from 24 subjects. Biomarkers and corresponding MAS grades from any 23 subjects formed the training dataset to learn a regression line by supervised multivariate regression analysis, and the remaining sample was used as the testing dataset to produce an evaluation score based on the input biomarkers and the learnt regression line. Each subject produced an evaluation score after 24 round of testing.

#### Final Assessment by Fusion of Both Models

Given different but complementary information from both models in terms of spasticity assessment, their fusion may ensure comprehensive and improved performance. Therefore, fusion of both models was implemented within the proposed framework, by combining their biomarkers through a supervised regression learning procedure. For each tested muscle of one subject, the TSRT from the lambda model, the four biomarkers (i.e., correlation coefficients of the angle, the angular velocity and the angular acceleration between the recorded curves and the re-constructed ones, and the MDF of the recorded angular acceleration) from the kinematic model were concatenated to form a five-dimensional feature vector. Similarly, a calibration procedure should be designed to interpret each feature vector as a meaningful evaluation score. We chose a SVR analysis for the calibration. The SVR is a version of support vector machine (SVM) for regression analysis which is based on the assumption of a linear regression function in a high dimensional feature space where the input data are mapped via a non-linear function (Basak et al., 2007). Compared with the ordinary least square regression, SVR can be used to fix both linear regression and non-linear regression tasks. Besides, it avoids multicollinearity problem between independent variables and reduce the impact of abnormal samples on model training thus offering more accurate model parameters. In this paper, we just briefly review the SVR analysis here as described in the literature (Basak et al., 2007; Ameri et al., 2014).

Assuming a set of training samples *D* = {(*x*_1_, *y*_1_), (*x*_2_, *y*_2_),...,(*x_L_*, *y_L_*)}, where *x_i_* ∈ *R^n^* represents input feature vectors and *y_i_* ∈ *R* represents the true MAS grades for all subjects. A non-linear projection ϕ(*x*) was applied for mapping the input data *x* to a higher dimensional space. Then the case of non-linear function *f* is described in the form as:

(1) $$f(x)=w^{T}\phi (x)+b$$

the SVR can be formulated as:

(2) $$\begin{array}{c} \underset{w,b,\xi_{i}^{-},\xi_{i}^{+}}{\min}\frac{1}{2}||w|{|}^{2}+C\sum_{i=1}^{L}\left(\xi_{i}^{-}+\xi_{i}^{+}\right)\\ s.t.f(x_{i})-y_{i}\leq \text{ε}+\xi_{i}^{-}\\ \;y_{i}-f(x_{i})\geq \text{ε}+\xi_{i}^{+}\\ \;\xi_{i}^{-}\geq 0,\;\xi_{i}^{+}\geq 0 \end{array}$$

where *C* is a properly chosen penalty factor, and $\xi_{i}^{-}$ and $\xi_{i}^{+}$ are slack variables representing upper and lower constraints on the outputs of the system. The Lagrangian multiplier method is used to solve the constrained optimization problem, and then the *f*(*x*) can be written in the following form:

(3) $$f(x)=\sum_{i=1}^{L}(\overset{⌢}{\alpha_{i}}-\alpha_{i})\kappa (x,\;x_{i})+b$$

where *κ*(*x_i_*, *x*) = *ϕ*(*x_i_*)*^T^ϕ*(*x*) is a radial basis function (RBF) kernel as follows:

(4) $$\kappa (x_{i},\;x)=\exp(-\frac{||x_{i}-x|{|}^{2}}{2\sigma^{2}}$$

The coefficients α_i_ and $\overset{⌢}{\alpha_{i}}$ in (3) are Lagrangian multiplier, and *b* is the bias constant determined by Karush-Kuhn-Tucker (KKT) conditions (Shevade et al., 2000).

In order to evaluate the spasticity for each subject, we similarly adopted a 24-fold leave-one-out cross validation. In this case, the input feature vectors ***x**_i_* and clinical MAS grades *y_i_* of any 23 subjects consisted of the training set, the remaining subject was used for testing to predict an evaluation score. During the training process, three important SVR parameters including penalty factor *C*, radius *ε* and a free parameter *σ* of RBF kernel needed to be determined toward optimal performance. Through some pretests, these parameters were empirically set at 30 for *C*, 0.09 for *ε* and 0.007 for *σ*. Finally, the well-trained SVR can be used as a tool to predict an evaluation score based on the input feature vector of any subject.

### Performance Evaluation and Statistical Analysis

For each of 24 subjects, three evaluation scores were obtained for muscle spasticity assessment from the lambda model, the kinematic model and their combination, respectively. In fact, each of those scores can be regarded as an estimate of the MAS because they were calibrated with the same scale. Therefore, mean square error (MSE) between the evaluation scores and the corresponding clinical MAS grades was calculated to evaluate the performance of each of the three methods for spasticity assessment.

(5) $$\text{MSE}=\frac{1}{L}\sum_{i=1}^{L}\left(Y_{i}-{\overset{⌢}{Y}}_{i}\right)$$

where *L* is the sample number, *Y_i_* is the clinical MAS grades and $\overset{⌢}{Y_{i}}$ represents the assessment indicators given by the three assessment model. There were five subgroups of subjects with five MAS grades rating 0, 1, 1+ (1.5), 2, and 3 in this study.

In order to examine capability of three assessment methods in discriminating subjects/muscles with different MAS grades, a two-way ANOVA was performed on the evaluation score with the assessment method considered as within-subject factor (three levels) and the MAS grade as the between-subject factor (five levels). *Post hoc* pairwise multiple comparisons with Bonferroni correction were used. The significance level was set to 0.05 for all analyses. All statistical analyses were completed using SPSS software (ver. 16.0, SPSS, Inc., Chicago, IL, United States).

## Results

**FIGURE 6:**
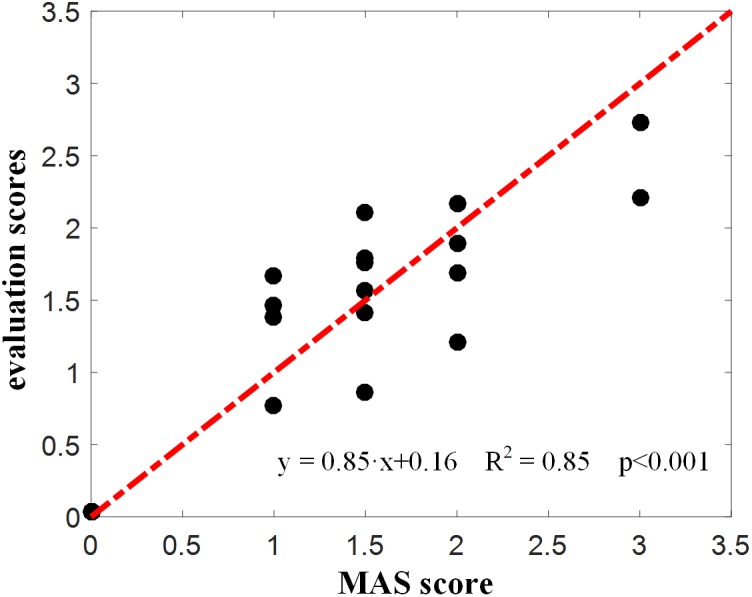
Evaluation scores derived from the lambda model for all subjects.

Figure 4 reports the experimental results from the lambda model. A representative example of the lambda model constructed for subject 1 was showed in Figure 4A. After excluding the outliers using 95% confidence region, the coefficient of determination *R*^2^ between angular velocity and the DSRT in linear regression analysis increased from 0.86 to 0.97. The DSRT was approximately linearly decreased with the increase in stretch angular velocity, which was verified by the regression line *y* = −0.277*x* + 46.765. Therefore, the obtained TSRT for this subject was 46.765. Figure 4B reports the distribution of TSRT values for all subjects categorized by MAS grades, where the healthy subjects had the same TSRT value of 120 degrees. It can be found that the TSRT was generally declined with increased grades of spasticity, showing a negative correlation between the MAS and the TSRT with a correlation coefficient of −0.93 for all subjects and −0.73 for subjects with spasticity. Subsequently, Figure 6 shows scatter plot of evaluation scores yielded by the lambda model versus the true MAS grades for all subjects. The evaluation score was regarded as a predicted MAS grades, and the MSE between those scores and corresponding MAS grades was 0.14. The linear regression analysis reported a high coefficient of determination *R*^2^ up to 0.84 between the evaluation scores and MAS grades.

**FIGURE 7:**
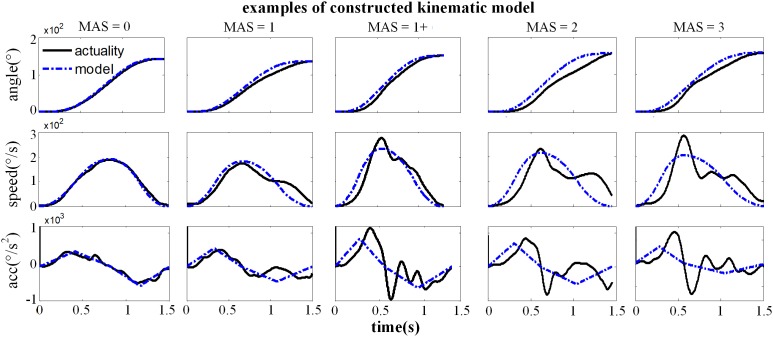
Representative examples for the constructed kinematic variables of a healthy subject (MAS = 0) and four subjects with spasticity (MAS = 1∼3), including the angle, speed and acceleration curve. The black solid line was the actual kinematic curve and the blue dashed line was the constructed intended curve.

**FIGURE 8:**
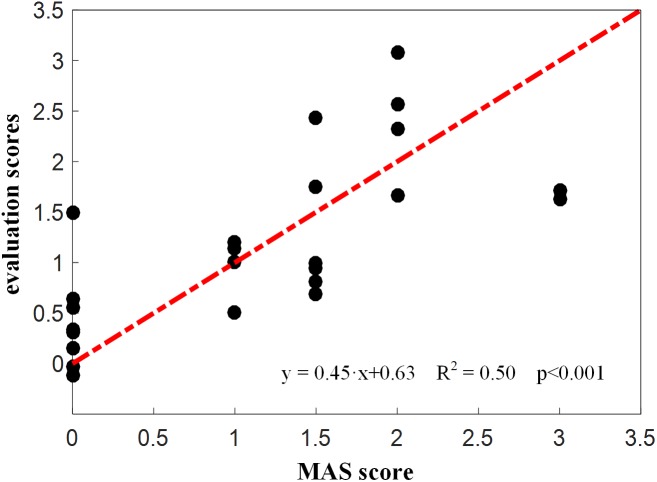
Evaluation scores derived from the kinematic model for all subjects.

Figure 7 showed the constructed kinematic curves for a healthy subject and four subjects with spasticity at different clinical MAS grades. From the subject 5 with a MAS grade of 1+, as a representative example, the kinematic curve showed a different pattern with noticeable deviations from the reference curve from healthy controls. This is always the case for all subjects with spasticity. A weak or strong correlation was presented between each of four biomarkers from the kinematic model and the MAS, with an absolute correlation coefficient ranging from 0.26 to 0.79, as exhibited in Table 3. Using the multivariate linear regression to calibrate the biomarkers from kinematic model, the final evaluation scores were reported in Figure 8 for all subjects. This method predicted greatly varied evaluation scores for subjects with the same MAS grade. Specifically, it failed to discriminate the subjects with a MAS grade of 3 by its predicted evaluation score. As a result, the MSE was 0.47 for the kinematic model, and the evaluation score derived from this model showed moderate goodness of fit (*R*^2^= 0.4990, *p* < 0.001) to the MAS grades.

**Table 3:** Correlation coefficients between each of biomarkers from the kenimatic model and the MAS grade.

Biomarkers		Correlation coefficient	*p*-value
Correlation coefficient	Acc^∗^	−0.79	*p*<0.001
	Velocity^∗^	−0.56	*p*<0.001
	Angle^∗^	−0.26	*p*>0.05
MDF	Acc	0.64	*p*<0.001

*Acc^∗^, velocity^∗^, angle^∗^: three biomarkers represent the correlation coefficients between the actual and reconstructed motion curves of angular acceleration, angular velocity and angle, respectively.*

**FIGURE 9:**
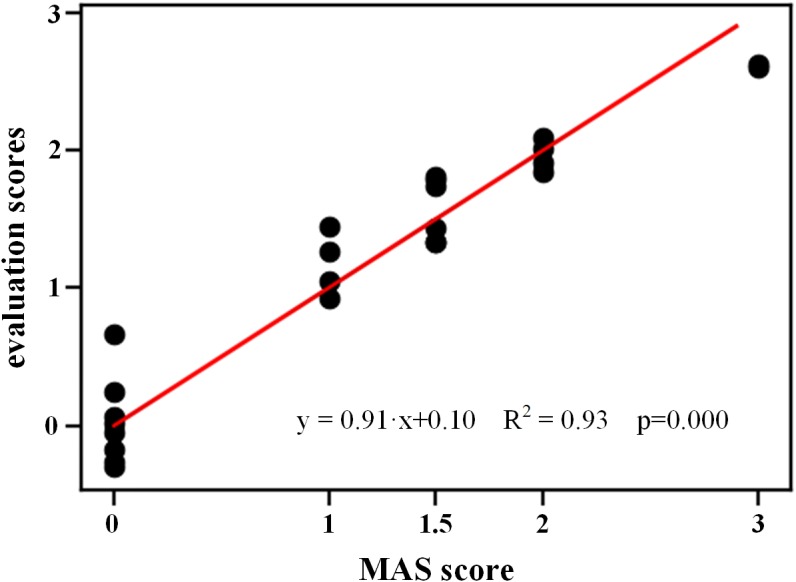
Evaluation scores derived from combination of both models using SVR for all subjects.

Figure 9 shows the evaluation scores obtained from fusion of both lambda and kinematic models using SVR for all subjects. The resultant evaluation scores appeared to positively correlate with the true MAS grades, with a regression line y = 0.91*x* + 0.10 whose goodness of fit was strong (*R^2^* = 0.93, *p* < 0.001), and a MSE of 0.059 was achieved. Furthermore, as compared with the single model, the combination of two models yielded the evaluation scores which are more distinguishable between any two MAS grades.

The ANOVA report no significant main effect of the evaluation method (*F* = 0.42, *p* > 0.05), but the spasticity degree (expressed as MAS grade) had a significant main effect on the evaluation scores (*F* = 53.6, *p* < 0.001). In addition, interaction between both factors was reported to be significant (*F* = 3.13, *p* < 0.01). Multiple pairwise comparisons further revealed that significant difference between any two MAS grades can be found except the comparisons between 1 and 1+, and between 1+ and 2, regardless of any method/model used.

## Discussion

This paper presents a novel framework for quantitative muscle spasticity assessment using supervised regression learning methods to mine combined sEMG and inertial data. The evaluation procedure consisted of a series of passive elbow stretch with different (subjectively determined) angular velocities, during which no specialized instrument was required for constant speed control. This simplified and convenient procedure is suitable for daily manipulation for it is truly in accordance with clinical routine and acceptable for both medical professionals and patients. The wearable design of the arm-bands embedded small, low-cost sEMG sensors and IMUs can especially ensure the acquisition of both neural and kinematic data in a user-friendly way. In addition, the use of regression learning, as a typical group of machine learning, enables establishment of an expert system without assistance of any clinician or other professional to make assessment decisions based on input sensory data. All these features ensure a great potential of the proposed framework in clinical, community and even home-based rehabilitation.

Within the proposed framework, two different models namely the lambda model and the kinematic model were tested, respectively. The performance of any single model was re-evaluated given sEMG and IMU data from low-cost, wearable sensors. The biomarkers extracted from the two models showed a weak to significant correlation with the MAS grades. After the calibration procedure, the evaluation scores obtained from each model had the same meanings as the corresponding clinical MAS grades, with significant correlation for lambda model (*R*^2^ = 0.84, *p* < 0.001) and strong correlation for kinematic model (*R^2^* = 0.50, *p* < 0.001) between them as well. All these findings were exactly consistent with those from previous studies (Feldman and Levin, 1995; Musampa et al., 2007; Calota et al., 2008). Such consistency not only confirmed the diagnostic efficiency of both models within the proposed framework in assessing muscle spasticity degrees, but also demonstrated that both models can work well under a specially designed protocol with simple testing procedures and essentially the same easily obtainable sensor apparatus for data recording. Therefore, under the proposed framework, the neurogenic component and non-neurogenic (mechanical) component of spasticity resistance can still be well-extracted and interpreted as potential quantitative evaluation descriptions using both models, respectively.

Please note that there are contradictory findings regarding the correlation between the lambda model and the MAS grade from the literature. Several studies have reported that this correlation was none or very poor (Levin et al., 2000; McGibbon et al., 2013), while others reported a good correlation (Kim et al., 2011). Consistent with the later report, our work exhibited a really good correlation with a coefficient of −0.93. The inconsistency between our study and others reporting no correlation can be mainly attributed into different technical details for data processing. For example, the TSRT of healthy individuals were conformably set at 120 due to the absence of typically evoked EMG associated with the spasticity. Exclusion of outliers was further implemented using a 95% confidence interval for the regression analysis, in order to overcome possible measurement errors given the portable sensors and manually controlled strength reflex. Both settings were specific to the lambda model conducted in our study. Another reason for explaining uncertain correspondence of the lambda model with the MAS is different way of their spasticity measurement. As discussed previously, the lambda model merely measured a certain aspect (i.e., neurogenic component) of the spasticity whereas the MAS emphasizes the spasticity-induced resistance to passive stretch of the muscle. If the neurogenic component becomes the primary contributor to muscle spasticity for a certain group of subjects, the lambda model is able to be highly correlated with the MAS, and otherwise, they may not agree very well. Both above-discussed reasons can also be used to explain result differences between the lambda model and kinematic model. It is suggested from the scatter plots (Figure 6, 7) that they may not correspond well with each other. In detail, the lambda model worked well in assessing muscles/subjects with MAS grades of 0 and 3, but gives indistinguishable judgments among MAS grades 1, 1+, and 2. By contrast, the kinematic model presents better performance in discriminating MAS grades 1, 1+ and 2, but fails to discriminate MAS grades of 0 and 3. All these findings are regarded as evidences suggesting advanced performance and comprehensive description of spasticity by information fusion of both models.

Given the complementary capabilities of both models in assessing spasticity, their fusion is also supported by the proposed framework. Beside two single-model evaluation methods, the third method employed the SVR working with all biomarkers derived from both models to produce a comprehensive evaluation score. By comparing the three evaluation methods, the method with two-model combination was found to outperform other two methods, in terms of the lowest MSE value. This confirms the necessity of information fusion toward improved performance. In fact, the stretch reflex resistance has been regarded to be formed by both the neurogenic and mechanical components of the spasticity, which can be well-described by the lambda model and the kinematic model, respectively. This provides a very straightforward reason for explaining improved performance when both models were considered by the proposed framework. Although each single model can be used alone for spasticity assessment with satisfactory performance, their combination further achieved improved performance with decreased MSE. Such a finding demonstrated feasibility and effectiveness of the proposed framework in combining both electrophysiological and kinematic information toward an advanced assessment of muscle spasticity. However, the ANOVA reported no significant difference between the three methods. The reason for explaining this could be attributed into the fact that all three evaluation methods within the proposed framework were calibrated into the same scale of the MAS. Despite of this, the ANOVA reported significant effect on the evaluation scores derived from these methods, indicating its capability of discriminating and quantifying different degrees of spasticity. In addition, comparing to the graded MAS grades, the evaluation method presented a more continuous distribution ranging from 0 to 3 (it was the maximum MAS for subjects recruited), which helps to enhance the accuracy and sensitivity of spasticity evaluation.

It is worth noticing that the proposed framework was applied to wearable low-precision sensors, and that an experiment protocol with free-hand manipulation was adopted in this study. As a result, the stretch velocities cannot be controlled and measured precisely. This would be expected to impact the efficacy of the proposed framework. In order to overcome these limitations, a series of trials with repeated passive elbow stretches were performed. It was also designed for ensuring sufficient data. Therefore, the proposed framework did not rely on data from any single trial, but mined these pooled low-precision data using machine learning algorithms to enhance the reliability and accuracy of spasticity evaluation. A representative example can be found in the lambda model. When quantifying a relationship between the dynamic stretch reflex threshold and angular velocity, a 95% confidence interval was designed in order to exclude the outliers. Finally, we found that the proposed framework for spasticity assessment works well on the low-precision data, yielding a high correlation to MAS grades, indicating the feasibility of using the simplified protocol in assessing muscle spasticity quantitatively. This also confirms the usability of the proposed framework.

However, there are still several limitations exist in this study. For all subjects, their MAS grades were taken as the regression target to calibrate and evaluate the computerized methods in this study. In another word, the MAS was regarded as the ground truth. However, the clinical MAS was a subjective evaluation with some well-known limitations, and thus it might not be considered to be absolutely accurate and trustable. For example, it is easy to misjudge the grade 1+ in the MAS. Therefore, the limitation in accuracy of the MAS might be the explanation of the failure in distinguishing the grade 1 and 1+, 1+ and 2 when implementing the proposed computerized method. In addition, due to the demand of sufficient data to ensure reliability of the evaluation method, a large number of repetitive trials are required. This protocol is a little time-assuming. The proposed framework employed relatively classic regression learning algorithms, many sophisticated learning algorithms such as deep learning may be beneficial to performance improvement, given recent development of machine learning techniques. Another key problem is the limited sample size used in this study. No subject with the maximal MAS grade of 4 was recruited due to inability of their tested muscles to complete the required experiment as a result of high stiffness. More samples would be benefit to enhance accuracy and reliability of the proposed framework according to supervised machine learning theory. Therefore, it is our expectation to enlarge more samples and to evolve the current work toward an expert system for quantitative spasticity assessment, suitable for clinical and family rehabilitation.

## Conclusion

The proposed evaluation framework is in line with clinical measurement practice, thus providing convenient, objective and promotable spasticity assessment method in clinics. Therefore, it provides feedback about therapy effect of patients, which helps in the design and adjustment of effective and individualized rehabilitation protocols and plays an important role in spasticity management and intervention for clinical or family rehabilitation.

## Ethics Statement

This study was approved by the Ethics Review Committee for Clinical Medical Research of First Affiliated Hospital of Anhui Medical University. All subjects offered informed and signed consent prior to any procedure of the experiment.

## Author Contributions

XuZ conceived the study and participated in the entire procedure of the study including experiments, data analyses, result interpretation, manuscript drafting and revisions. XT participated in the data analyses and interpretation, and performed substantial revisions of the manuscript. XiZ conducted experiments and participated in manuscript drafting. XG conceived the study and participated in result interpretation and manuscript revisions. XiC and XuC participated in data analysis and interpretation, and manuscript revisions. XuC also coordinated the study. All authors approved the final version of the manuscript.

## Conflict of Interest Statement

The authors declare that the research was conducted in the absence of any commercial or financial relationships that could be construed as a potential conflict of interest.
